# Increasing pentose phosphate pathway flux enhances recombinant protein production in *Pichia pastoris*

**DOI:** 10.1007/s00253-016-7363-5

**Published:** 2016-03-28

**Authors:** Justyna Nocon, Matthias Steiger, Teresa Mairinger, Jonas Hohlweg, Hannes Rußmayer, Stephan Hann, Brigitte Gasser, Diethard Mattanovich

**Affiliations:** Department of Biotechnology, BOKU, University of Natural Resources and Life Sciences Vienna, Muthgasse 18, 1190 Vienna, Austria; Austrian Centre of Industrial Biotechnology, Muthgasse 11, 1190 Vienna, Austria; Department of Chemistry, BOKU, University of Natural Resources and Life Sciences Vienna, Muthgasse 18, 1190 Vienna, Austria

**Keywords:** Recombinant protein, Pentose phosphate pathway, Metabolic flux analysis, Zwf1, Sol3

## Abstract

Production of heterologous proteins in *Pichia pastoris* (syn. *Komagataella* sp.) has been shown to exert a metabolic burden on the host metabolism. This burden is associated with metabolite drain, which redirects nucleotides and amino acids from primary metabolism. On the other hand, recombinant protein production affects energy and redox homeostasis of the host cell. In a previous study, we have demonstrated that overexpression of single genes of the oxidative pentose phosphate pathway (PPP) had a positive influence on recombinant production of cytosolic human superoxide dismutase (hSOD). In this study, different combinations of these genes belonging to the oxidative PPP were generated and analyzed. Thereby, a 3.8-fold increase of hSOD production was detected when glucose-6-phosphate dehydrogenase (*ZWF1*) and 6-gluconolactonase (*SOL3*) were simultaneously overexpressed, while the combinations of other genes from PPP had no positive effect on protein production. By measuring isotopologue patterns of ^13^C-labelled metabolites, we could detect an upshift in the flux ratio of PPP to glycolysis upon *ZWF1* and *SOL3* co-overexpression, as well as increased levels of 6-phosphogluconate. The substantial improvement of hSOD production by *ZWF1* and *SOL3* co-overexpression appeared to be connected to an increase in PPP flux. In conclusion, we show that overexpression of *SOL3* together with *ZWF1* enhanced both the PPP flux ratio and hSOD accumulation, providing evidence that in *P. pastoris* Sol3 limits the flux through PPP and recombinant protein production.

**Electronic supplementary material:**

The online version of this article (doi:10.1007/s00253-016-7363-5) contains supplementary material, which is available to authorized users.

## Introduction

Production of heterologous proteins has long been shown to exert a metabolic burden on the producing host cell (Bentley et al. 1990; Glick 1995). This phenomenon has been observed in all main classes of host organisms (bacteria, yeasts, mammalian cells), typically leading to a reduction of the maximum specific growth rate and of biomass yield. It is part of a stress reaction to protein overproduction (for a review see Mattanovich et al. (2004)). Yeasts are well established as host cells for production of recombinant proteins, and among them *Pichia pastoris* (syn. *Komagataella* sp.) is widely used today (Gasser et al. 2013). Decreased biomass production in protein producing *P. pastoris* strains has often been observed (e.g., (Dragosits et al. 2009; Heyland et al. 2010; Jorda et al. 2012).

Metabolic burden has been attributed to the rerouting of metabolites (mainly nucleotides and amino acids) from cellular pathways to the recombinant product. Besides that, the extra demand of energy and reducing power has been discussed to be responsible for metabolic burden (Heyland et al. 2011). Similarly, Dragosits et al. (2009) observed a decreased metabolic flux to biomass formation while TCA cycle flux increased in a strain producing an antibody Fab fragment compared to a non-producing control strain, thus pointing at an increased energy demand of the producing strain. In recombinant *Escherichia coli*, the increase of ATP production by reversion of the phosphoenolpyruvate carboxykinase reaction led to higher production of GFP (Kim et al. 2012). Flores et al. (2004) engineered the pentose phosphate pathway (PPP) of *E. coli* to alleviate the metabolic burden of recombinant protein overproduction. Overexpression of *E. coli* glucose-6-phosphate dehydrogenase (*zwf*) compensated for the growth deficit imposed by production of a proinsulin fusion peptide. Further metabolic engineering approaches to improve recombinant protein production in *E. coli* were reviewed recently (Liu et al. 2015; Mahalik et al. 2014).

Following a systems-based approach, we have recently shown that overexpression of enzymes in the oxidative branch of the PPP improved the production of human superoxide dismutase (hSOD) in *P. pastoris* (Nocon et al. 2014). Of the four first reactions of PPP, overexpression of 6-phosphogluconolactonase (*SOL3*) had the strongest positive influence, leading to 40 % increase in hSOD productivity. Also, glucose-6-phosphate dehydrogenase (*ZWF1*) showed a weak positive effect, while 6-phosphogluconate dehydrogenase (*GND2*) and D-ribulose-5-phosphate 3-epimerase (*RPE1*) had no influence on protein production. In *Saccharomyces cerevisiae*, glucose-6-phosphate dehydrogenase is regulated at the enzyme activity level by the NADPH/NADP^+^ ratio (Zubay 1988) while 6-phosphogluconolactonase is controlled at the transcriptional and translational level (Castelli et al. 2011; Zampar et al. 2013). These data are supported by the finding that gradual increase of the NADPH demand led to upregulation of *SOL3* and *GND1*, but not of *ZWF1* (Celton et al. 2012). Contrary, we showed recently that in *P. pastoris ZWF1* is upregulated while *SOL3* expression is not changed in glucose-limited conditions or on methanol media (Prielhofer et al. 2015), both conditions that lead to an increase of PPP flux (Baumann et al. 2010; Russmayer et al. 2015a). Thus, comparative conclusions on PPP regulation should be drawn cautiously between these two yeasts.

While lactonase reactions can occur spontaneously, albeit at low rate, they have been observed as a rate-limiting step in the oxidation of sugars (Buchert and Viikari 1988). For instance initial xylonate production by overexpression of xylose dehydrogenase in *S. cerevisiae* was higher when also D-xylonolactonase was co-overexpressed (Nygard et al. 2014; Toivari et al. 2012).

These data lead to the hypothesis that not only one enzyme is limiting the PPP rate, and thus that co-overexpression of *ZWF1* and *SOL3*, and possibly also other genes of the oxidative PPP branch may further enhance the positive effect on recombinant protein production. Therefore, we have evaluated the combined overexpression of these genes on their impact on PPP flux and hSOD production in *P. pastoris*.

## Materials and Methods

### Strains and vectors

All *P. pastoris* strains used in this study, apart from the wild-type strain X-33 (Invitrogen), were based on the strain intracellularly producing human superoxide dismutase (Marx et al. 2009). The pPM2 expression vectors overexpressing single *P. pastoris* genes *ZWF1*, *SOL3*, *GND2*, and *RPE1* under the control of strong, constitutive glyceraldehyde-3-phosphate dehydrogenase (GAP) promoter were described previously (Nocon et al. 2014). For generation of double overexpression vectors, a second expression cassette, consisting of GAP promoter, gene, and terminator was inserted into vectors already containing one expression cassette using the restriction sites *Apa*I, *Mre*I, and *Age*I that generate overlapping ends. For this purpose, the expression cassettes of *SOL*3 and *RPE1* were excised using *Mre*I and *Age*I and ligated into *ZWF1* or *GND1* expression vectors linearized by *Apa*I and *Mre*I. The vectors were linearized in the genome integration locus (either 3′-region of *AOX1* or the 5′-region of *ENO1*) and integrated into the genome of electrocompetent *P. pastoris* cells (Gasser et al. 2013). Hygromycin (HphMX) or Geneticin (KanMX) resistance were used as selection markers. Positive transformants were selected on YPD containing 500 μg/mL Zeocin and 500 μg/mL G418 or 200 μg/mL hygromycin. Additionally, to combine the first two PPP steps, a *P. pastoris* strain overexpressing *SOL3* was transformed with the *ZWF1* overexpression vector after recycling of the selection marker with Cre recombinase (Marx et al. 2008).

### Determination of gene copy numbers with quantitative PCR (qPCR)

Genomic DNA was extracted from overnight cultures using Wizard® Genomic DNA Purification Kit (Promega) according to the manufacturer’s protocol. The purified DNA was quantified with NanoDop2000c (Thermo Scientific). Gene copy numbers were determined with quantitative real-time PCR using SensiMix SYBR Kit (QT605-05, Bioline, UK) and a Rotor Gene 6000 (Qiagen, DE) real-time PCR cycler. Primers are listed in Table 1. All samples (genomic DNA diluted to 5 ng μL^−1^) were analyzed in tri- or quadruplicates. The occurrence of primer dimers and the purity of the PCR-product were checked by melting curve analysis. Relative gene copy numbers were determined with the comparative quantification method of the Rotor Gene software using strain SOD as calibrator. For each gene, the fluorescence values were related to the calibrator strain SOD which is expected to contain one copy of each analyzed gene. The resulting values were then normalized to the gene *ARP1* for each strain, which is also present in single copy in the genome of *P. pastoris*.

**Table 1:** Sequences of primers used for RT-qPCR

Target gene	Forward primer	Reverse primer
*ZWF1*	CGACGTTTTGGTGGGTCAAT	ACACCTTCCCACCTTTCTGT
*SOL3*	TGAACATCACCTTCCCAGTGT	AAGGTGGCTTGGGAGAATCT
*RPE1*	AGGCTTTGGTGGACAGAAGT	ATCGGCTGCTACTCCTATGG
*GND2*	TCCCATTTCCCTGACACCAA	CCACCTGGCATCAAAGAAGG
*ARP1*	GTCCAGCATAAACACGCCG	CAGTGGGAAAAACCCACGAA

### Determination of transcript levels by reverse transcription quantitative PCR (RT-qPCR)

Total RNA was isolated from overnight cultures. RNA isolation, complementary DNA (cDNA) synthesis and measurement of messenger RNA (mRNA) transcript levels using RT-qPCR was performed as described in Stadlmayr et al. (2010). The isolated RNA was quantified with NanoDrop2000c (Thermo Scientific), and its quality was assessed using agarose gel electrophoresis before it was used for cDNA synthesis. Equal amounts of cDNA were used for real-time PCR determination of relative transcript levels. Quantitative real-time PCR was performed as described for gene copy number determination and analyzed using the comparative quantification method of the Rotor Gene software. The transcript levels of each gene were normalized to the actin-related housekeeping gene *ARP1* as internal control; the strain SOD served as the calibrator strain for each relative transcript level determination with the delta-delta Ct method (Pfaffl 2001).

### Superoxide dismutase (hSOD) quantification

The strains were cultivated at 25 °C in 10 mL YPD supplemented with 2 mM CuCl_2_ and 0.02 mM ZnSO_4_ in 100 mL shake flasks without baffles. The cultures were grown for 48 h and fed three times in 12 h intervals with 100 μL 50 g L^−1^ glucose.

At the end of the cultivation, 1 mL of culture was harvested by centrifugation. The cell pellet was resuspended in 500 μL of extraction buffer (20 mM Tris-HCl pH 8.2, 5 mM EDTA, 0.1 % Triton X-100, 7 mM ß-mercaptoethanol, 1 mM CuCl_2_, 0.01 mM ZnSO_4_, SIGMA*FAST*™ protease inhibitor cocktail (Sigma)) and mechanically disrupted with glass beads (diameter 0.5 mm) on FastPrep® in 3 cycles of 20 s at 6.5 m s^−1^ with 5 min resting time on ice. The hSOD concentrations were measured by ELISA and correlated to total protein concentration (measured with Coomassie Protein Assay).

### ^13^C Metabolic Flux Analysis

For metabolic flux analysis, cells were grown in 50 mL YNB medium (3.4 g L^−1^ YNB w/o amino acids and ammonium sulfate, 10 g L^−1^ (NH_4_)_2_SO_4_, 400 mg L^−1^ biotin) with 2.5 g L^−1^ 1,6-^13^C-labeled glucose (CortecNet, Voisins-le-Bretonneux, France) as a single C-source. The cultures were grown in wide neck shake flasks without baffles at 25 °C. The cells were inoculated at OD_600_ = 0.03 and grown for 20 h until mid-exponential phase (around OD_600_ = 1). Glucose uptake and extracellular metabolites were determined in cultures grown on naturally labeled glucose. Glucose, ethanol, acetate, and arabitol were quantified from supernatants by HPLC as described in Pflugl et al. (2012).

For the analysis of intracellular metabolites, the cells were rapidly sampled into 60 % methanol at −30 °C and filtered through cellulose acetate filters using a vacuum pump (Russmayer et al. 2015b). Briefly, the cell pellet on the filter was transferred to precooled tubes and stored at −80 °C until extraction. The metabolites were extracted by adding 4 mL of 75 % ethanol at 85 °C to the frozen cell pellets and incubating it for 3 min at 85 °C. The samples were rapidly cooled and the extracts separated by centrifugation.

^13^C labeling patterns of selected sugar phosphates were analyzed according to Chu et al. (2015) employing an Agilent 7890B gas chromatograph in combination with an Agilent 7200 GC-QTOFMS system. The mass accuracy of the system was <5 ppm. Prior to analysis, a two-step derivatization was performed online on a GERSTEL DualRail MultiPurposeSampler (MPSII, GERSTEL, Germany). Data evaluation involved isotope interference correction for the contribution of heavy isotopes from the derivatization agent and the native molecule itself, employing the software “Isotope correction toolbox- ICT” (Jungreuthmayer et al. 2016). Isotopologue information on glyceraldehyde-3-phosphate (GAP), 2-phosphoglycerate (2PG), 3-phosphoglycerate (3PG), dihydroxyacetone phosphate (DHAP), ribulose-5-phosphate (Rul5P), fructose-6-phosphate (F6P), sedoheptulose-7-phosphate (S7P), phosphoenolpyruvate (PEP), ribose-5-phosphate (Ri5P), and erythrose-4-phosphate (E4P) was employed for further flux calculation. In addition to the metabolites described in Chu et al. (2015), ^13^C labeling information on alanine (ALA) was included and obtained by using the analysis method described above extracting the following masses: 234.1340, 235.1374, 236.1407, and 237.1441 with a mass extraction window of 100 ppm. The mass distribution vectors determined for the mentioned metabolites in the different strains can be found in Supplementary Table S1.

### Flux calculation

Specific growth rates, glucose uptake rates, and metabolite secretion rates were determined during the exponential growth phase between 17 and 23 h after inoculation. The biomass composition of *P. pastoris* wild-type strain X-33 was determined previously (Carnicer et al. 2009). Flux calculations were performed with OpenFLUX using standard settings and applying the Monte Carlo approach for sensitivity analysis (Quek et al. 2009). The stoichiometric model (provided in Supplementary Table S2) was based on a previously published *P. pastoris* model of the central carbon metabolism (Baumann et al. 2010; Nocon et al. 2014) and was limited to the upper glycolysis and pentose phosphate pathway. The model was constrained for glucose uptake, biomass and arabitol secretion, and fitted against the mass distribution vectors of GAP, 3PG, 2PG, DHAP, Rul5P, F6P, S7P, PEP, Ri5P, E4P, and ALA.

## Results

Based on predictions from a genome scale metabolic model, we demonstrated recently that the overexpression of single genes of the pentose phosphate pathway can have a positive effect on production of recombinant proteins (Nocon et al. 2014). The majority of the predictions were related to the upper, oxidative branch of the PPP, namely the genes *ZWF1*, *SOL3*, *GND2*, and *RPE1* (Table 2). This work prompted the obvious question whether combined overexpression of more than one of these genes may have a synergistic effect on protein production. We therefore first created double overexpressing clones of *ZWF1* and *SOL3* (the first and second step of oxidative PPP, strain ZS), *GND2* and *RPE1* (the third and fourth step, strain GR), as well as *ZWF1* and *RPE1* (the first and the last step, strain ZR), and then attempted quadruple overexpression (strain ZSGR). Therefore, additional copies of these genes under control of the strong glycolytic P_*GAP*_ promoter were introduced into the *P. pastoris* genome.

**Table 2:** Genes in the upper, oxidative branch of the pentose phosphate pathway

Gene name	ORF name*	Description	Reaction
*ZWF1*	PIPA08178 PAS_chr2-1_0308	Glucose-6-phosphate dehydrogenase; catalyzes the first PPP step: the NADP^+^ dependent oxidation of glucose-6-phosphate	Glucose-6-P+NADP^+^→6-phosphogluconolactone + NADPH+H^+^
*SOL3*	PIPA04435 PAS_chr3_1126	6-Phosphogluconolactonase; catalyzes the second PPP step opening the lactone ring	6-Phosphogluconolactone→6-phosphogluconate
*GND2*	PIPA03124 PAS_chr3_0277	6-Phosphogluconate dehydrogenase (decarboxylating); catalyzes the NADP^+^ dependent oxidative decarboxylation of 6-phosphogluconate	6-phosphogluconate+NADP^+^→ribulose-5-P+ CO_2_+NADPH+H^+^
*RPE1*	PIPA03251 PAS_chr3_0441	D-Ribulose-5-phosphate 3-epimerase; catalyzes a reaction connecting the oxidative to the non-oxidative part of the PPP	Ribulose-5-P↔xylulose-5-P

*PIPA* ORF name in strain DSMZ70382, *PAS* ORF name in strain GS115

**Fig. 1:**
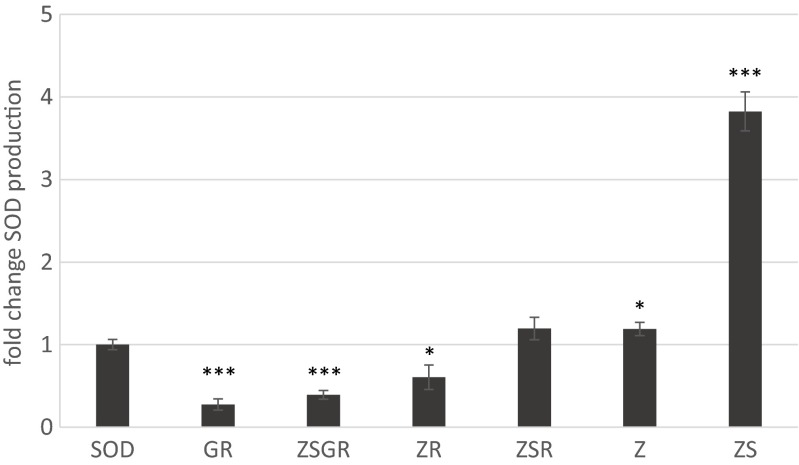
The effect of overexpression of multiple PPP genes on production of recombinant human superoxide dismutase (*hSOD*). *SOD* = *P. pastoris* strain producing hSOD, *Z* = *ZWF1*, *S* = *SOL3*, *G* = *GND2*, *R* = *RPE1*. Changes of hSOD yield (μg hSOD per mg of total extracted protein) relative to the SOD strain are depicted. *Error bars* represent standard errors of the means of 6 to 10 individual clones, respectively. Significance levels of the difference of each strain to the parental SOD strain is indicated as follows: ***p < 0.01, **p < 0.05, and *p < 0.1

To study the effect of combined overexpression of PPP genes on the production of recombinant hSOD, 6 to 10 clones of each strain were tested. The parental SOD strain produced on average 28.4 mg hSOD per gram soluble protein (meaning that approx. 3 % of cellular protein are hSOD). We first evaluated the accumulation of hSOD in strains overexpressing two PPP genes. While the GR construct led to a reduction of hSOD expression, the combination of the first two steps (*ZWF1* and *SOL3*) resulted in exceptional, three- to fourfold increase of hSOD accumulation in the cells (Fig. 1). Overexpression of *RPE1* together with *ZWF1* or *GND2* led to a decrease in produced hSOD levels, yielding 60 and 50 % of the original hSOD production respectively which possibly points to a metabolic imbalance in these strains. From these data, we conclude that a combination of the first two PPP reactions is highly beneficial to recombinant protein production while combinations including the further downstream reactions have detrimental effects.

To further analyze the effect of the *ZWF1* and *SOL3* combination on protein production, both genes were transformed into a GR strain, yielding ZSGR strains. Similarly a ZSR strain was constructed. Accumulation of hSOD was drastically lower in the ZSR clones compared to the ZS strains, and even decreased below the levels of the unmodified SOD strain in the ZSGR strain, indicating that the total balance of expression levels of the PPP genes is a major factor determining optimum production of recombinant protein.

**Fig. 2:**
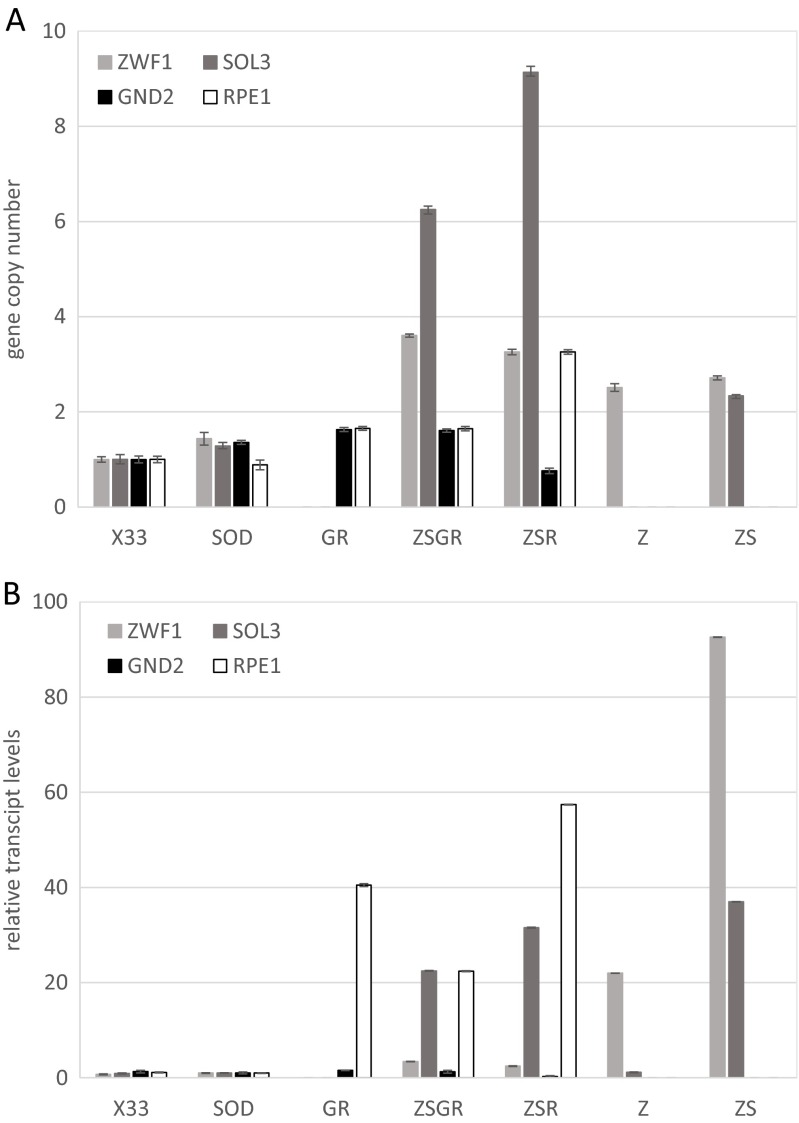
Gene copy numbers and mRNA expression levels of genes involved in the pentose phosphate pathway: *ZWF1*, *SOL3*, *GND2*, and *RPE1*. Quantitative real-time PCR was used to determine relative differences between strains. The *ARP1* gene was used for normalization. *Error bars* indicate standard deviation of three to four technical replicates. The copy numbers of all four genes were analyzed in X-33 and SOD; only the overexpressed genes were determined in the PPP engineered strains. **a** Copy numbers of PPP genes in the engineered. strains relative to the wild-type control X-33. X-33 is assumed to contain one copy of each gene. **b** Relative mRNA expression levels of PPP genes compared to the SOD strain

Quantitative PCR was used to determine the gene copy numbers in selected engineered strains (Fig. 2A). The differences between wild-type (X-33) and hSOD production strain were minimal. As the *P. pastoris* genome contains only one paralog of each of these genes (De Schutter et al. 2009), a gene copy number of one was pre-assigned for the four analyzed genes, *ZWF1*, *SOL3*, *GND2*, and *RPE1*, in X-33. As supposed, strain SOD contains also only one copy of each strain. *ZWF1* was found in 2–3 copies in all the modified strains except for ZSGR with 4 copies. The number of *SOL3* copies was the highest in ZSGR strains (9 copies). Only one additional copy was integrated in ZS double overexpressing strains. In all strains overexpressing *RPE1* the respective gene copy number was 2–3. One additional copy of *GND2* was detected in GR and ZSGR. Altogether, this proves that one or more additional copies of the engineering targets under control of P_GAP_ were integrated into the *P. pastoris* genome, thus enabling constitutive overexpression of the respective genes with different transcriptional levels.

RT-qPCR was applied to measure changes in transcriptional levels of the four target genes (Fig. 2B). Transcript levels of the four PPP genes were arbitrarily set to 1 for the SOD strain. In comparison, the wild-type strain X-33 showed only minor differences in expression of the analyzed genes. In the strains where additional copies of *SOL3* under control of the strong GAP promoter were introduced, *SOL3* transcript levels were strongly enhanced, being on average 30-fold higher compared to the production strain SOD irrespective of the *SOL3* gene copy number. Also, for *RPE1*, very high transcript levels were observed in the modified strains, showing a positive correlation with increasing gene copy number. *ZWF1* expression was predominantly enhanced in the clones overexpressing only *ZWF1*, and in *SOL3* strains transformed with an additional copy of the *ZWF1* overexpression vector. Clones transformed with the double vectors (ZWF1/SOL3) had increased, but low expression of *ZWF1*, however. The GR and ZSGR strains containing one additional *GND2* copy had slightly increased *GND2* expression levels (approximately twofold higher). This low increase can be attributed to the fact that the native expression level of *GND2* is much higher than of all other PPP genes: it is in the same range as of *TDH3* (encoding glyceraldehyde-3-phosphate dehydrogenase), as determined by DNA microarrays (Prielhofer et al. 2015; Rebnegger et al. 2014). Thus, an increase in *GND2* transcript levels by overexpression under control of the P_GAP_ promoter is expected to be less effective than of the other genes studied here.

**Fig. 3:**
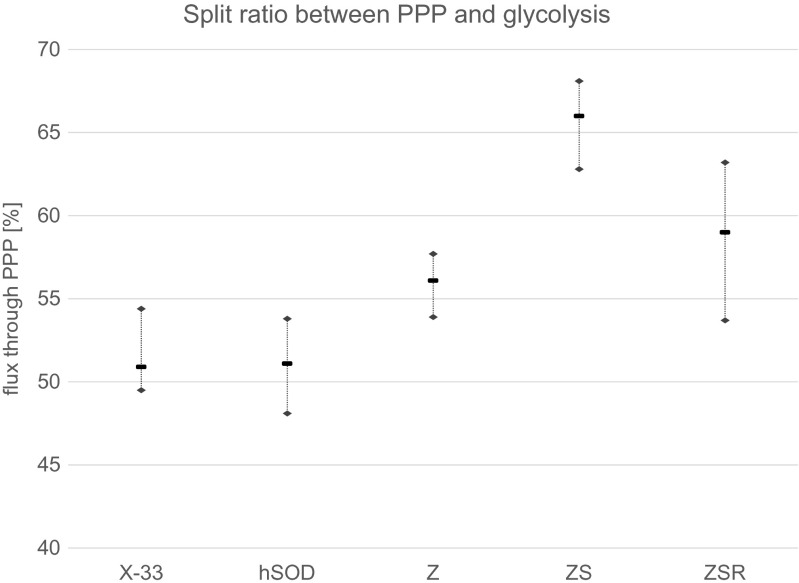
The ratio of pentose phosphate pathway flux to glycolysis in X-33 wild type, hSOD production strain, and three modified strains: Z, ZS, and ZSR. The flux ratio is calculated from the fluxes v2 and v15 in the flux model (Supplementary Table S2), and the optimal solution is shown as a *horizontal black bar*. A 95 % confidence interval, which is highlighted by flanking diamonds, was calculated using the Monte Carlo approach implemented in OpenFLUX for sensitivity analysis

The flux ratios at the PPP/glycolysis branching point were determined with ^13^C-metabolic flux analysis. The selected clones were cultivated on minimal medium supplemented with ^13^C-labeled glucose as a single carbon source. Glucose carrying ^13^C in positions 1 and 6 was chosen as a substrate for its better suitability for resolving glycolysis and PPP fluxes in a single experiment (M. Antoniewicz, personal communication; Mairinger et al. (2015)). Especially, F6P carries the information about the flux branch between PPP and glycolysis and was used as one of the 11 intracellular metabolites to determine the flux ratio between PPP and glycolysis. Fractions of PPP flux at the PPP/glycolysis branch point are shown in Fig. 3. The PPP split ratio did not change in the SOD strain compared to the X-33 strain confirming the observation made with uniformly labeled glucose (Nocon et al. 2014). Overexpression of *ZWF1* increased the PPP flux ratio by about 6 % while double overexpression of *ZWF1* and *SOL3* lead to a 15 % increase of the flux split ratio towards PPP. Additional overexpression of *RPE1* decreased the PPP flux, which matches with the decrease of hSOD accumulation in the ZSR clones. In summary, these data clearly indicate that overall hSOD production levels are strongly correlated with an increase in net-PPP flux.

**Fig. 4:**
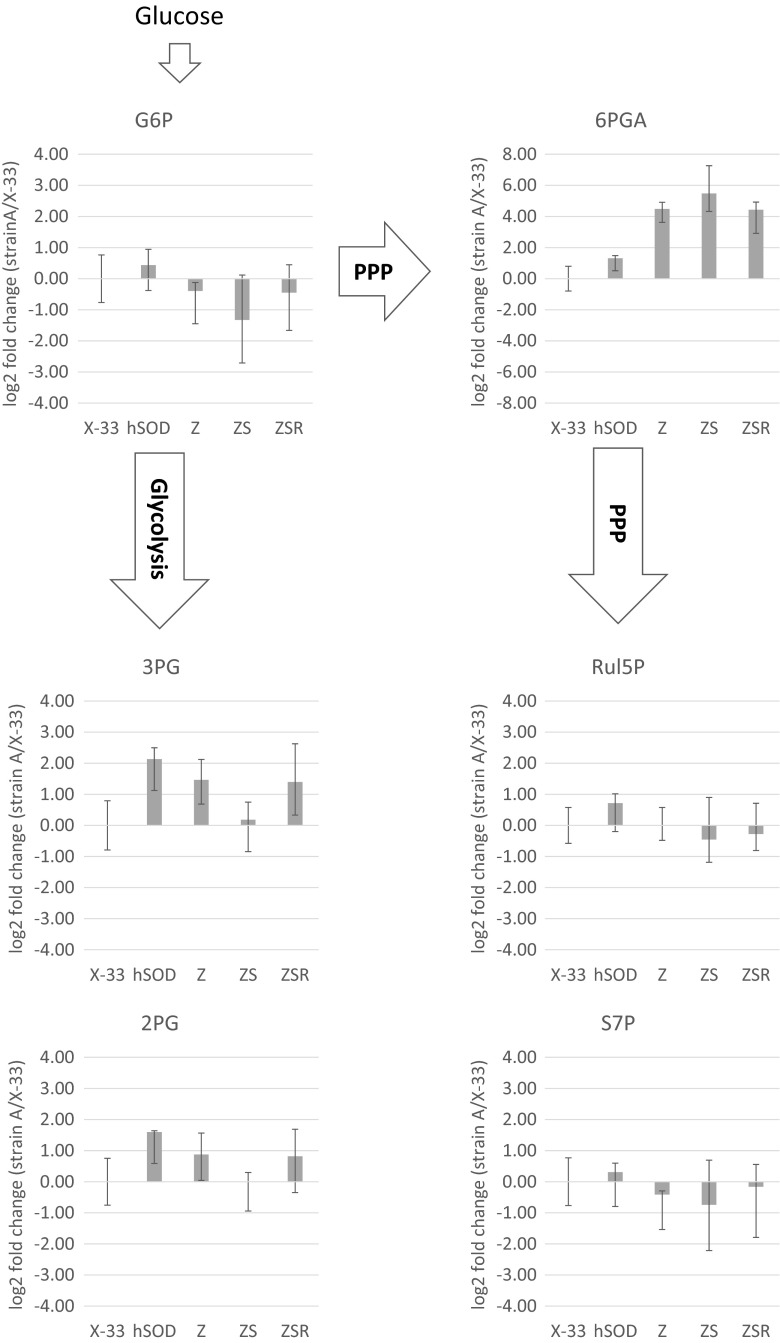
Changes in metabolite levels in X-33 wild type, SOD production strain, and three modified strains: Z, ZS, and ZSR. The log_2_ fold changes of six different metabolites are displayed comparing the respective strain to the X-33 wild type. The six metabolites are glucose-6-phosphate (*G6P*), 6-phosphogluconate (*6PGA*), ribulose-5-phosphate (*Rul5P*), sedoheptulose-7-phosphate (*S7P*), 3-phosphoglycerate (*3PG*), and 2-phosphoglycerate (*2PG*). The metabolites are arranged according to their occurrence in glycolysis or PPP. The *blue bars* correspond to the log_2_ fold change calculated from the median of three biological replicates. *Error bars* are calculated from the extreme values of the three biological replicates

Furthermore, the changes in intracellular metabolite levels of the different strains were analyzed using semi-quantitative metabolite data from the ^13^C metabolic flux analysis. The relative changes of intracellular metabolites were calculated and are displayed for six metabolites of glycolysis and PPP in Fig. 4. A remarkable change can be observed for 6-phosphogluconate (6PGA). It should be noted that GC-MS cannot distinguish between the open sugar acid and the respective lactone ring, so that these values are sum parameters of 6-phospho-glucono-δ-lactone (6PGDL) and 6PGA. A significant increase of this metabolite was found for strains overexpressing *ZWF1* and further increased by the overexpression of *SOL3*. In the strain carrying the double overexpression *ZWF1* and *SOL3*, the amount of 6PGA had a 45 times higher concentration than the X-33 wild-type strain. In the ZSR strain, the additional overexpression of *RPE1* reduces the 6PGA levels slightly compared to the ZS strain. Interestingly, no other metabolites of the PPP showed significant changes (Fig. 4). Exemplarily, Rul5P and S7P are displayed but also E4P and R5P did not have altered metabolite levels.

In the lower glycolysis part, the levels of 3PG and 2PG were elevated in the SOD strain. Overexpression of *ZWF1* reduced this value slightly, and the double expression of *ZWF1* and *SOL3* re-established the levels of the wild-type strain. Interestingly, in the ZSR strain, again, a higher level is found comparable to the ZS strain.

## Discussion

Enhancing recombinant protein production by overexpression of PP pathway genes has been postulated by metabolic modeling and was also verified experimentally (Nocon et al. 2014). Especially, *SOL3* overexpression had a markedly positive effect which was attributed to its tight control in yeast and its potential role as rate limiting step of oxidative PPP (Castelli et al. 2011; Zampar et al. 2013). The present work provides evidence that in *P. pastoris*, Sol3 (6-phosphogluconolactonase) is rate limiting for PPP flux and recombinant protein production, as, in a synergistic fashion, overexpression of *SOL3* together with *ZWF1* enhanced both the PPP flux ratio and hSOD accumulation. In other words, the positive effect of opening the PPP flux by overexpressing the initial step *ZWF1*, which produces reduced NADPH, can only be fully exploited when the following lactonase reaction is also deregulated and enhanced. Lactonase has also been demonstrated to facilitate rapid opening of the lactone ring during production of xylonate from xylose in recombinant *S. cerevisiae* (Nygard et al. 2014). Given the low native expression level of *SOL3* in *P. pastoris* (Rebnegger et al. 2014), it appears plausible that overexpression of *ZWF1* alone leads to a rate limitation of the lactonase reaction and consequently to an accumulation of 6PGDL which will limit the glucose-6-phosphate dehydrogenase reaction by product inhibition. Co-overexpression of *SOL3* alleviates this rate limitation at the lactonase reaction and thus enables to make full use of the potential flux enhancement by *ZWF1* overexpression. The measured accumulation of 6PGA indicates that while the first bottleneck has been opened and the oxidation reaction by *ZWF1* is more efficient with co-overexpression of *SOL3*, there is still another yet unidentified bottleneck further downstream. While the positive synergy between *ZWF1* and *SOL3* is obvious, it is less intuitive to explain the obvious negative effect of further co-expression of the downstream genes *GND2* and *RPE1*. The obvious metabolic imbalance caused by this multiple co-overexpression will require further research for a full explanation.

Single overexpression of *GND2* decreased hSOD production, differently to all other upper PPP genes (Nocon et al. 2014). Strikingly, overexpression of *GND2* even had a negative impact on hSOD productivity when the whole PPP flux is increased in strains with combined overexpression. *GND2* is among the most abundantly expressed genes of *P. pastoris* (Rebnegger et al. 2014), indicating that its transcription is probably not rate limiting. Further overexpression may imbalance the PPP flux leading to a negative impact on product formation. The strain co-overexpressing *RPE1* with *ZWF1* and *SOL3* had decreased PPP flux ratio and hSOD production, compared to the ZS double overexpression strain. Ribulose 5-phosphate 3-epimerase is a potentially reversible reaction. Overexpression may lead to a shift of the concentrations of the reactants which may explain the decrease of PPP flux and protein production.

The intracellular concentrations of 6GPA (actually the sum of 6PGDL and 6PGA) increased markedly in the PPP engineered strains. Being the products of the reaction catalyzed by Zwf1 and the following lactone ring opening by Sol3, an increased concentration of these metabolites comes not as a surprise; however, the order of magnitude exceeded our expectations. The fact that Sol3 overexpression further enhanced 6GPA levels provides additional evidence that this is a rate limiting step. The accumulation, however, points to a further limitation downstream which we could not identify in this work. In addition, hSOD overproduction led to increased accumulation of lower glycolysis intermediates which was reverted back to wild-type levels in the ZS double overexpression strain. The present data do not allow a conclusion on the reasons for metabolite accumulation in lower glycosylation, but we note that ZS overexpression allows the cells to overcome this metabolic shift.

Finally, we were interested whether the enhanced PPP flux would also positively affect biomass growth. However, no difference in maximum specific growth rates and biomass yield coefficients could be observed between the different hSOD producing strains. Thus, the enhanced PPP flux did not alleviate the observed growth defect of the SOD strain (Marx et al. 2009) while it enhanced hSOD production.

In conclusion, we could further support a prediction made with the aid of a genome-scale metabolic model, namely that an increased flux through the PPP would enhance recombinant protein production. We found further evidence that Sol3 (6-phosphogluconolactonase) is, in synergy with Zwf1 (glucose 6-phosphate dehydrogenase), the rate limiting step of the oxidative PPP. Overexpression of *ZWF1* alone did not enhance the PPP flux, while the co-overexpression with *SOL3* enabled an increased PPP flux and three- to fourfold higher hSOD production. This value is higher than that achieved with *SOL3* overexpression alone (Nocon et al. 2014), indicating that also Zwf1 is rate limiting, but the effect of its overexpression can only be exploited when the produced lactone ring is efficiently opened by synergistic overproduction of Sol3. Overexpression of the later steps (*GND2*, *RPE1*) obviously imbalanced the PPP, as the flux ratio decreased. Further research will be needed to clarify the flux controlling role of these two enzymes.

Reduced NADPH is an important cofactor for production of many metabolites. It will be interesting to investigate in the future if, and to which extent, *ZWF1*/*SOL3* overexpressing strains of *P. pastoris* and other production hosts are suitable platforms for such production processes.

## Electronic Supplementary Material

ESM 1 (PDF 115 kb)
