# Supplementary material for: Increasing pentose phosphate pathway flux enhances recombinant protein production in Pichia pastoris
Source: Appl Microbiol Biotechnol. 2016 Mar 28;100:5955–63. doi: 10.1007/s00253-016-7363-5 (PMC4909809; doi:10.1007/s00253-016-7363-5)
Supplement: Supplementary file 1 — (PDF 115 kb) [file 253_2016_7363_MOESM1_ESM.pdf]

Applied Microbiology and Biotechnology

Supplementary File:

Increasing pentose phosphate pathway flux enhances recombinant protein production in *Pichia pastoris*

Justyna Nocon[1]*, Matthias Steiger[1,2]*, Teresa Mairinger[2,3], Jonas Hohlweg[1,2], Hannes Rußmayer[1], Stephan Hann[2,3], Brigitte Gasser[1,2§], Diethard Mattanovich[1,2]

*equally contributing authors

[1]Department of Biotechnology, BOKU – University of Natural Resources and Life Sciences Vienna, Muthgasse 18, 1190 Vienna, Austria

[2]Austrian Centre of Industrial Biotechnology, Muthgasse 11, 1190 Vienna, Austria

[3]Department of Chemistry, BOKU - University of Natural Resources and Life Sciences Vienna, Muthgasse 18, 1190 Vienna, Austria

[§]Corresponding author:

Brigitte Gasser

Department of Biotechnology, University of Natural Resources and Life Sciences

Muthgasse 18, 1190 Vienna, Austria

Tel. +43-1-47654-6813

Email: brigitte.gasser@boku.ac.at

Supplementary Table S1: Measured mass distribution values (MDV) of the strains X-33, hSOD, Z, ZS and ZSR cultivated on ^{13}C 1, 6 glucose are shown in the right column. The deviations of 3 biological replicates are shown in the middle column and the MDVs of the OpenFLUX fitting yielding the lowest residual error are shown in the right column.

	X33				hSOD				Z				ZS				ZSR			
	Median		Deviation	simulated	Median		Deviation	simulated	Median		Deviation	simulated	Median		Deviation	simulated	Median		Deviation	simulated
Ala_M	0.114	±	0.003	0.170	0.121	±	0.021	0.172	0.115	±	0.011	0.190	0.135	±	0.001	0.230	0.134	±	0.029	0.200
Ala_M1	0.868	±	0.001	0.811	0.852	±	0.022	0.809	0.860	±	0.014	0.791	0.847	±	0.007	0.753	0.841	±	0.026	0.782
Ala_M2	0.018	±	0.003	0.019	0.026	±	0.003	0.019	0.025	±	0.003	0.019	0.018	±	0.008	0.016	0.024	±	0.004	0.018
Ala_M3	0.000	±	0.000	0.000	0.000	±	0.000	0.000	0.000	±	0.000	0.000	0.000	±	0.000	0.000	0.000	±	0.000	0.000
GAP_M	0.162	±	0.021	0.170	0.183	±	0.027	0.172	0.155	±	0.019	0.190	0.195	±	0.023	0.230	0.191	±	0.034	0.200
GAP_M1	0.789	±	0.007	0.811	0.767	±	0.024	0.809	0.788	±	0.021	0.791	0.756	±	0.006	0.753	0.757	±	0.017	0.782
GAP_M2	0.049	±	0.017	0.019	0.043	±	0.006	0.019	0.052	±	0.004	0.019	0.044	±	0.021	0.016	0.043	±	0.013	0.018
GAP_M3	0.000	±	0.001	0.000	0.007	±	0.002	0.000	0.005	±	0.003	0.000	0.005	±	0.005	0.000	0.009	±	0.006	0.000
2PG_M	0.218	±	0.021	0.170	0.131	±	0.112	0.172	0.198	±	0.046	0.190	0.226	±	0.019	0.230	0.219	±	0.063	0.200
2PG_M1	0.782	±	0.037	0.811	0.869	±	0.112	0.809	0.771	±	0.068	0.791	0.749	±	0.049	0.753	0.749	±	0.089	0.782
2PG_M2	0.000	±	0.025	0.019	0.000	±	0.000	0.019	0.030	±	0.021	0.019	0.024	±	0.016	0.016	0.032	±	0.028	0.018
2PG_M3	0.000	±	0.007	0.000	0.000	±	0.000	0.000	0.001	±	0.002	0.000	0.001	±	0.019	0.000	0.000	±	0.005	0.000
DHAP_M	0.220	±	0.015	0.231	0.226	±	0.031	0.232	0.201	±	0.023	0.218	0.235	±	0.023	0.247	0.246	±	0.045	0.254
DHAP_M1	0.738	±	0.012	0.750	0.743	±	0.024	0.749	0.746	±	0.013	0.763	0.724	±	0.014	0.737	0.721	±	0.039	0.729
DHAP_M2	0.043	±	0.007	0.019	0.030	±	0.008	0.018	0.053	±	0.010	0.019	0.040	±	0.009	0.016	0.033	±	0.006	0.017
DHAP_M3	0.000	±	0.001	0.000	0.000	±	0.000	0.000	0.000	±	0.000	0.000	0.000	±	0.004	0.000	0.000	±	0.002	0.000
3PG_M	0.139	±	0.017	0.170	0.077	±	0.065	0.172	0.124	±	0.029	0.190	0.169	±	0.015	0.230	0.129	±	0.061	0.200
3PG_M1	0.861	±	0.017	0.811	0.923	±	0.065	0.809	0.876	±	0.029	0.791	0.831	±	0.015	0.753	0.871	±	0.061	0.782
3PG_M2	0.000	±	0.000	0.019	0.000	±	0.000	0.019	0.000	±	0.000	0.019	0.000	±	0.000	0.016	0.000	±	0.000	0.018
3PG_M3	0.000	±	0.000	0.000	0.000	±	0.000	0.000	0.000	±	0.000	0.000	0.000	±	0.000	0.000	0.000	±	0.000	0.000
E4P_M	0.000	±	0.000	0.060	0.000	±	0.000	0.061	0.000	±	0.000	0.073	0.000	±	0.000	0.016	0.000	±	0.000	0.075
E4P_M1	0.948	±	0.070	0.882	0.923	±	0.161	0.880	1.000	±	0.012	0.865	1.000	±	0.000	0.953	1.000	±	0.060	0.871
E4P_M2	0.052	±	0.070	0.057	0.077	±	0.161	0.058	0.000	±	0.012	0.060	0.000	±	0.000	0.031	0.000	±	0.060	0.053
E4P_M3	0.000	±	0.000	0.001	0.000	±	0.000	0.001	0.000	±	0.000	0.001	0.000	±	0.000	0.000	0.000	±	0.000	0.001
E4P_M4	0.000	±	0.000	0.000	0.000	±	0.000	0.000	0.000	±	0.000	0.000	0.000	±	0.000	0.000	0.000	±	0.000	0.000

Rl5P_M	0.073 ± 0.015	0.010	0.100 ± 0.030	0.010	0.084 ± 0.013	0.010	0.092 ± 0.010	0.010	0.113 ± 0.037	0.010
Rl5P_M1	0.856 ± 0.054	0.949	0.891 ± 0.057	0.949	0.822 ± 0.034	0.949	0.863 ± 0.040	0.949	0.848 ± 0.056	0.949
Rl5P_M2	0.031 ± 0.035	0.041	0.000 ± 0.056	0.041	0.057 ± 0.034	0.041	0.002 ± 0.019	0.041	0.007 ± 0.018	0.041
Rl5P_M3	0.040 ± 0.032	0.001	0.000 ± 0.014	0.001	0.026 ± 0.017	0.001	0.041 ± 0.035	0.001	0.017 ± 0.011	0.001
Rl5P_M4	0.000 ± 0.004	0.000	0.009 ± 0.005	0.000	0.012 ± 0.012	0.000	0.000 ± 0.007	0.000	0.014 ± 0.001	0.000
Rl5P_M5	0.000 ± 0.002	0.000	0.000 ± 0.002	0.000	0.000 ± 0.000	0.000	0.000 ± 0.000	0.000	0.000 ± 0.000	0.000
R5P_M	0.088 ± 0.010	0.107	0.103 ± 0.016	0.093	0.097 ± 0.009	0.134	0.101 ± 0.010	0.085	0.116 ± 0.032	0.140
R5P_M1	0.841 ± 0.051	0.845	0.890 ± 0.054	0.868	0.828 ± 0.039	0.828	0.827 ± 0.038	0.824	0.813 ± 0.053	0.824
R5P_M2	0.062 ± 0.046	0.047	0.000 ± 0.050	0.038	0.054 ± 0.033	0.037	0.052 ± 0.031	0.089	0.042 ± 0.027	0.036
R5P_M3	0.008 ± 0.022	0.001	0.000 ± 0.010	0.001	0.016 ± 0.015	0.001	0.019 ± 0.020	0.002	0.026 ± 0.020	0.001
R5P_M4	0.001 ± 0.007	0.000	0.007 ± 0.004	0.000	0.005 ± 0.005	0.000	0.000 ± 0.004	0.000	0.003 ± 0.002	0.000
R5P_M5	0.000 ± 0.000	0.000	0.000 ± 0.001	0.000	0.000 ± 0.000	0.000	0.000 ± 0.000	0.000	0.000 ± 0.001	0.000
F6P_M	0.060 ± 0.009	0.035	0.079 ± 0.015	0.035	0.089 ± 0.031	0.046	0.118 ± 0.011	0.077	0.115 ± 0.017	0.052
F6P_M1	0.369 ± 0.015	0.359	0.359 ± 0.007	0.359	0.414 ± 0.039	0.403	0.476 ± 0.015	0.470	0.435 ± 0.037	0.420
F6P_M2	0.550 ± 0.026	0.578	0.551 ± 0.024	0.577	0.427 ± 0.081	0.525	0.394 ± 0.030	0.434	0.438 ± 0.021	0.504
F6P_M3	0.019 ± 0.013	0.028	0.008 ± 0.005	0.028	0.070 ± 0.043	0.026	0.010 ± 0.006	0.018	0.012 ± 0.028	0.024
F6P_M4	0.002 ± 0.003	0.001	0.000 ± 0.000	0.001	0.000 ± 0.000	0.000	0.002 ± 0.016	0.000	0.000 ± 0.016	0.000
F6P_M5	0.000 ± 0.001	0.000	0.002 ± 0.005	0.000	0.000 ± 0.002	0.000	0.000 ± 0.000	0.000	0.000 ± 0.000	0.000
F6P_M6	0.000 ± 0.001	0.000	0.000 ± 0.000	0.000	0.000 ± 0.000	0.000	0.000 ± 0.000	0.000	0.000 ± 0.000	0.000
S7P_M	0.067 ± 0.011	0.039	0.081 ± 0.017	0.041	0.063 ± 0.012	0.052	0.089 ± 0.013	0.010	0.098 ± 0.028	0.050
S7P_M1	0.682 ± 0.023	0.684	0.680 ± 0.032	0.688	0.701 ± 0.030	0.691	0.739 ± 0.005	0.767	0.718 ± 0.003	0.733
S7P_M2	0.246 ± 0.023	0.261	0.240 ± 0.036	0.258	0.216 ± 0.027	0.244	0.170 ± 0.008	0.212	0.183 ± 0.027	0.207
S7P_M3	0.006 ± 0.009	0.016	0.000 ± 0.007	0.013	0.013 ± 0.006	0.013	0.000 ± 0.003	0.010	0.000 ± 0.000	0.010
S7P_M4	0.000 ± 0.000	0.000	0.000 ± 0.000	0.000	0.006 ± 0.006	0.000	0.000 ± 0.000	0.000	0.000 ± 0.001	0.000
S7P_M5	0.000 ± 0.000	0.000	0.000 ± 0.000	0.000	0.000 ± 0.001	0.000	0.001 ± 0.002	0.000	0.000 ± 0.001	0.000
S7P_M6	0.000 ± 0.000	0.000	0.000 ± 0.000	0.000	0.000 ± 0.000	0.000	0.000 ± 0.000	0.000	0.000 ± 0.000	0.000
S7P_M7	0.000 ± 0.000	0.000	0.000 ± 0.000	0.000	0.000 ± 0.001	0.000	0.000 ± 0.000	0.000	0.000 ± 0.000	0.000
PEP_M0	0.173 ± 0.017	0.170	0.175 ± 0.019	0.172	0.158 ± 0.017	0.190	0.191 ± 0.009	0.230	0.196 ± 0.027	0.200
PEP_M1	0.804 ± 0.031	0.811	0.813 ± 0.021	0.809	0.836 ± 0.028	0.791	0.800 ± 0.037	0.753	0.804 ± 0.028	0.782
PEP_M2	0.000 ± 0.013	0.019	0.009 ± 0.005	0.019	0.006 ± 0.010	0.019	0.009 ± 0.016	0.016	0.000 ± 0.004	0.018
PEP_M3	0.023 ± 0.004	0.000	0.004 ± 0.002	0.000	0.000 ± 0.001	0.000	0.000 ± 0.014	0.000	0.000 ± 0.003	0.000

Supplementary Table S2:
Stoichiometric model of glycolysis and pentose phosphate pathway for fitting of the ^{13}C flux measurements in OpenFLUX. The model is based on the model published by Baumann *et al*. 2010 BMC Syst Biol 4:141

RxnID	Reaction Equation	Carbon Transition	Type
R1	GLC_EX + ATP = G6P + ADP	abcdef + X = abcdef + X	F
R2	G6P = F6P	abcdef = abcdef	F
R3	F6P + ATP = FBP + ADP	abcdef + X = abcdef + X	F
R4	FBP = F6P + Pi	abcdef = abcdef + X	F
R5	FBP = DHAP + G3P	abcdef = abc + def	F
R6	DHAP + G3P = FBP	abc + def = abcdef	F
R7	DHAP = G3P	abc = abc	F
R8	G3P = DHAP	abc = abc	F
R9	G3P + ADP + NAD = PG3 + ATP + NADH	abc + X + X = abc + X + X	F
R10	PG3 = PG2	abc = abc	F
R11	PG2 = PG3	abc = abc	F
R12	PG2 = PEP	abc = abc	F
R13	PEP = PG2	abc = abc	F
R14	PEP = PYR	abc = abc	F
R15	G6P + NADP = RUL5P + CO2 + NADPH	abcdef + X = bcdef + a + X	F
R16	RUL5P = XYL5P	abcde = abcde	F
R17	XYL5P + ADP = XYL + ATP	abcde + X = abcde + X	F
R18	XYL + NADH = AROL + NAD	abcde + X = abcde + X	F
R19	RUL5P = RIB5P	abcde = abcde	F
R20	XYL5P + RIB5P = SED7P + G3P	abcde + fghij = fgabcde + hij	F
R21	SED7P + G3P = XYL5P + RIB5P	abcdefg + hij = cdefg + abhij	F
R22	SED7P + G3P = E4P + F6P	abcdefg + hij = defg + abchij	F
R23	E4P + F6P = SED7P + G3P	abcd + efghij = efgabcd + hij	F
R24	XYL5P + E4P = F6P + G3P	abcde + fghi = abfghi + cde	F
R25	F6P + G3P = XYL5P + E4P	abcdef + ghi = abghi + cdef	F
R26	F6P = F6Pex		B
R27	PYR = PYRex		B
R28	AROL = AROLex		B
R29	CO2 = CO2ex		B
R30	RIB5P = RIB5Pex		B
R31	G6P = G6Pex		B
R32	E4P = E4Pex		B
R33	PYR = ALA	abc = abc	S
R34	PG3 = SER	abc = abc	S
R35	SER = GLY + MTHF	abc = ab + c	S

excludedMetabolites
GLC_EX
ADP
ATP
NAD

NADH
NADP
NADPH
AROLex
CO2ex
Pi
PYRex
F6Pex
RIB5Pex
G6Pex
E4Pex

simulatedMDVs
ALA#111
G3P#111
PG2#111
DHAP#111
PG3#111
E4P#1111
RUL5P#11111
RIB5P#11111
F6P#111111
SED7P#1111111
PEP#111

inputSubstrates
GLC_EX
